# Kinetic energy efficiency of single ventricle and TCPC using 4D flow MRI

**DOI:** 10.1186/1532-429X-17-S1-Q97

**Published:** 2015-02-03

**Authors:** Alejandro Roldán-Alzate, Sylvana García-Rodríguez, Petros V Anagnostopoulos, Shardha Srinivasan, Christopher J Francois

**Affiliations:** 1 Radiology, University of Wisconsin, Madison, WI, USA; 2 Cardio-Thoracic Surgery, University of Wisconsin, Madison, WI, USA; 3 Pediatric Cardiology, University of Wisconsin, Madison, WI, USA

## Background

Altered hemodynamics in total cavopulmonary connection (TCPC), a palliation of single ventricle defects, results in long-term complications, such as decreased exercise capacity, arrhythmia, and ventricular failure [1]. Non-invasive hemodynamic evaluation of TCPC has been an important clinical challenge. Several studies have tried to understand and predict specific flow features using a combination of patient-specific MRI data and computational tools to develop more realistic numerical and physical models. Most numerical studies have based their analyses of TCPC efficiency on energy loss calculations, but assumptions such as rigid walls and idealized flow conditions might affect accuracy hindering clinical applicability [2]. The purpose of this study was to calculate kinetic energy (KE) from *in vivo* 4D Flow MRI velocity measurements, in the TCPC and single ventricle for assessing efficiency of the system.

## Methods

**Figure 1:**
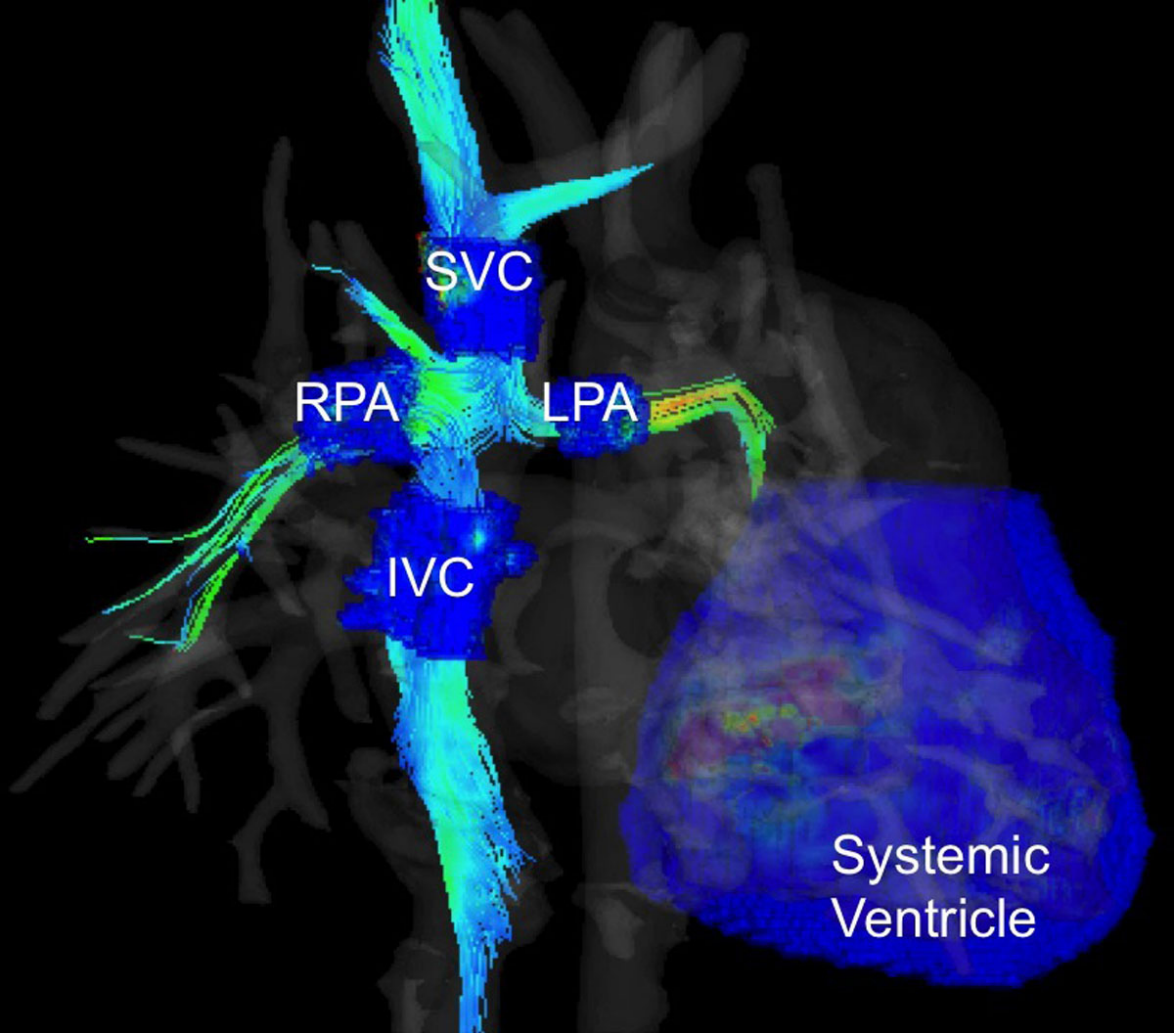
Visualization of flow and kinetic energy in an extracardiac TCPC and its systemic ventricle obtained from 4D flow MRI.

4D Flow MRI was performed in 6 TCPC patients and 6 healthy subjects following an IRB-approved protocol. 4D flow MRI (PC VIPR) imaging parameters were: imaging volume: 32x32x24cm, 1.25mm acquired isotropic spatial resolution, TR/TE=6.4/2.2ms, VENC = 200cm/s (extra-cardiac) and 100cm/s (atrio-pulmonary) [3]. Vessel segmentation was performed (MIMICs, Materialise, Leuven, Belgium) from PC angiograms; visualization and quantification were performed in EnSight (CEI, Apex, NC). Two calculations were considered: 1) the energy efficiency of the TCPC, and thus in the pulmonary circulation, and 2) the energy efficiency of the systemic ventricle, thus evaluating the systemic circulation. For the first, KE_in_ (KE_IVC_ + KE_SVC_) was compared to KE_out_ (KE_RPA_ + KE_LPA_) as %KE loss = (KE_in -_ KE_out_)/KE_in_. For the second calculation, ventricular energy efficiency was defined as the ratio of aortic flow and systemic ventricular KE (Q_Ao_/KE_SV_) (Figure 1).

## Results

**Figure 2:**
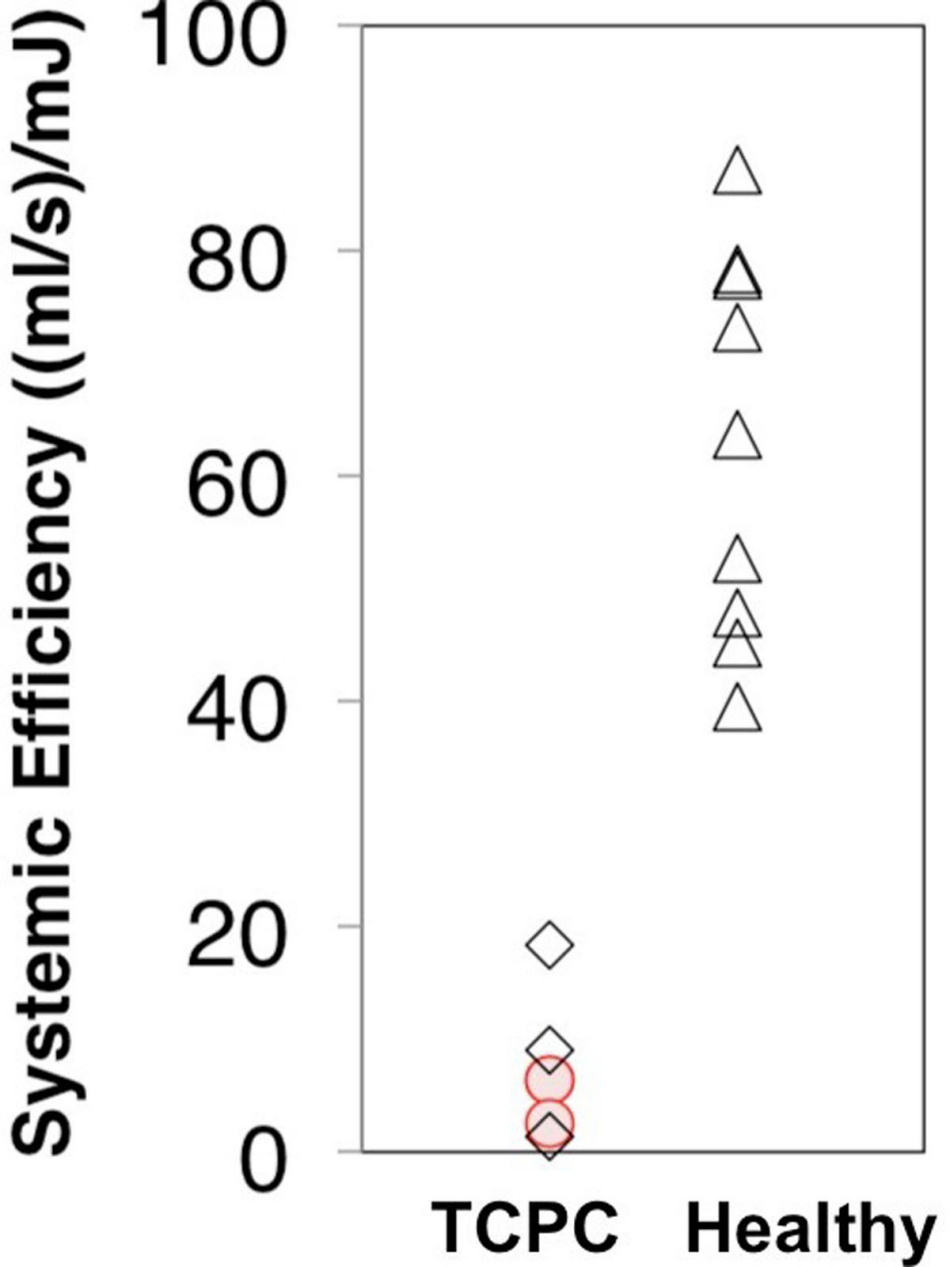
Systemic efficiency in healthy (Δ) and TCPC subjects. Single left ventricle (red circles) and single right ventricle (black diamonds).

Kinetic energy loss in the TCPC was 31 ± 20%. Figure 2 compares systemic efficiency in TCPC and healthy subjects. Systemic efficiency was lower in TCPC patients (7.6 ± 6.1 (ml/s)/mJ) than that in healthy volunteers (63 ± 17 (ml/s)/mJ)). Within the TCPC group, patients with single right ventricle had higher (9.5 ± 8.5 (ml/s)/mJ) that those with left single ventricle (5.2 ± 2.2 (ml/s)/mJ).

## Conclusions

As expected, TCPC patients had less systemic efficiency; however, high variability was observed in both groups suggesting the need for larger sample studies. Successful implementation of in vivo 4D Flow MRI provides a powerful non-invasive surveillance tool that has the potential to allow the clinician to follow the performance of TCPC over time, identify potential hemodynamic deterioration and intervene in an asymptomatic phase to increase the life span of the operation.

## Funding

American Heart Association - Scientist Development Grant (Alejandro Roldán-Alzate).
